# Interactive Effects of Rarefaction and Surface Roughness on Aerodynamic Lubrication of Microbearings

**DOI:** 10.3390/mi10020155

**Published:** 2019-02-25

**Authors:** Yao Wu, Lihua Yang, Tengfei Xu, Haoliang Xu

**Affiliations:** 1 State Key Laboratory for Strength and Vibration of Mechanical Structures, Xi’an Jiaotong University, Xi’an 710049, China; nealjackman@stu.xjtu.edu.cn (Y.W.); xtf1992@stu.xjtu.edu.cn (T.X.); xuhaoliang1988@stu.xjtu.edu.cn (H.X.); 2 Shaanxi Key Laboratory of Environment and Control for Flight Vehicle, Xi’an 710049, China; 3 School of Aerospace Engineering, Xi’an Jiaotong University, Xi’an 710049, China

**Keywords:** gaseous rarefaction effects, fractal surface topography, modified Reynolds equation, aerodynamic effect, bearing characteristics

## Abstract

The aerodynamic lubrication performance of gas microbearing has a particularly critical impact on the stability of the bearing-rotor system in micromachines. Based on the Duwensee’s slip correction model and the fractal geometry theory, the interactive effects of gas rarefaction and surface roughness on the static and dynamic characteristics were investigated under various operation conditions and structure parameters. The modified Reynolds equation, which governs the gas film pressure distribution in rough bearing, is solved by employing the partial derivative method. The results show that high values of the eccentricity ratio and bearing number tend to increase the principal stiffness coefficients significantly, and the fractal roughness surface considerably affects the ultra-thin film damping characteristics compared to smooth surface bearing.

## 1. Introduction

The microfluidic devices are widely used in many applications such as ultra-precision machine tool spindles, inertial navigation system (INS), medical devices, and hard disk drives (HDDs) in micro-electro-mechanical systems (MEMS). Since the development of microsystems engineering technology, gas journal microbearing has been generally preferred over the electromagnetic bearing and rolling bearing owing to its advantages of simple structure, high rotary accuracy, high running speed, low friction power loss, and wide working temperature range [1,2,3]. The real surface of a mechanical part produced by various machining and finish operations is composed of a large number of distributed peaks and valleys. The lubricating film thickness between the surfaces of shaft and bearing has continually decreased, which results in the increase of the roughness heights that are of the same order of magnitude as the minimum clearance gap. Thus, the assumptions that the gas flow is typically treated as a continuum flow with no slip boundary condition and the bearing surface roughness is considered negligible in classical fluid mechanics have to be revised at microscales [4,5,6,7]. The interaction between rarefaction and surface roughness in microbearing can influence the reliability and operational efficiency of micro rotating machinery obviously, so the gas journal microbearing performance should be comprehensively analyzed.

In order to accurately predict the effects of surface roughness and rarefaction on the bearing characteristics, many researchers devoted numerous research efforts to study the complicated flow behaviors at very small clearances over the last few decades. In consideration of rarefaction effects in ultra-thin gas film lubrication, the Knudsen number *K_n_* is utilized to describe the rarefied gas flow, which is defined as the ratio of molecular mean free path *λ*_0_ to the characteristic length of gas film thickness. Burgdorfer [8], Hsia et al. [9], and Mitsuya [10], based on the slip velocity boundary condition, derived the classical first order, second order, and 1.5 order slip models in the slider/disk interface for HDDs to take into account the effect of gas rarefaction. Fukui and Kaneko [11,12] developed a Poiseuille flow rate database for a wide Knudsen number range to modify the compressible Reynolds type equation including thermal creep flow and accommodation coefficient from linearized Boltzmann equation. In order to account for the effect of surface roughness, Christensen and Tønder [13,14] presented a stochastic model of hydrodynamic lubrication for finite width journal bearing in which they considered the lubricant film thickness as a stochastic process. The operating characteristics of bearing was theoretically analyzed with roughness pattern, nominal geometric features, and statistical properties by surface averaging techniques. Via linear transformation of random matrices, the Gaussian or non-Gaussian distribution of surface heights were generated by Patir [15] using the prescribed autocorrelation functions and frequency density functions. Patir and Cheng [16] further derived the average Reynolds equation suitable for various roughness structure and discussed the effect of roughness on mean hydrodynamic pressure, mean viscous friction, and mean bearing inflow in finite slider bearings. The average flow model of Patir and Cheng was extended by Tripp [17], in which the statistical expectation of flow factors were calculated with a perturbation expansion of the film pressure. The results showed that the flow factors are closely correlated with roughness parameters. White et al. [18] introduced the transverse sinusoidal roughness pattern to study the influence of surface roughness on steady-state pressure profiles of wedge bearing by variable grid implicit finite difference method and found that the load capacity could be decreased to a limiting value at higher bearing numbers. For the applications of perturbation technique and mapping function, Li et al. [19] studied the effects of roughness orientations and rarefaction on static performance of magnetic recording systems. The results demonstrated that the flow factors changed with the orientation angle and Peklenik number, and the effect of moving surface on surface characteristics is more significant than that of the stationary surface. Turaga et al. [20] proposed the stochastic finite method to solve Reynolds equation and obtained the static and dynamic performance of hydrodynamic journal bearings with the longitudinal, transverse and isotropic roughness pattern. Naduvinamani et al. [21] established the surface roughness by a stochastic random variable with nonzero mean, variance and skewness, and the average Reynolds equations were adopted to analyze the performance of porous step-slider bearings with Stokes couple stress fluid. Zhang et al. [22,23] presented the modified Reynolds equation by including fractal roughness effect and velocity slip boundary condition and concluded that the flow behaviors in gas-lubricated journal microbearings was appreciably affected by Knudsen number, bearing number and fractal dimension. The coupling effects of non-Newtonian micropolar fluids and roughness on the dynamic characteristics of plane slider bearings were investigated by Lin et al. [24] on the basis of the microcontinuum theory and Christensen stochastic roughness model. They indicated that the transverse roughness serves to somewhat increase bearing dynamic property, whereas the longitudinal roughness would tend to decrease the dynamic coefficients. Jao et al. [25] examined the influences of surface roughness and anisotropic slips on hydrodynamic lubrication of journal bearings. They described the lubricant flow in rough bearing surface by the product of flow factors and flow in nominal film thickness, and also identified that boundary slip reduced the effect of surface roughness. Kalavathi et al. [26] reported a generalized Reynolds equation for finite porous slider bearing with both longitudinal and transverse roughness. The authors showed the surface roughness enhanced the pressure distribution and load carrying capacity while the permeability parameter diluted the load. Quiñonez [27] utilized the linear superposition of perturbation method and Flourier transformation to provide a solution for the flow characteristics of wide exponential land slider bearings with rough surfaces. The results were in good agreement with the cases of sinusoidal and single Gaussian dent. The linear perturbation method was used by Wang et al. [28] to solve the unsteady Reynolds equation for rough aerostatic journal bearings during the iterative process, and the dynamic performance was obtained by taking into account the interactions of journal rotation and surface waviness. However, likely due to the nonlinear and complexity of dynamic flow behavior, previous papers were mainly focusing on steady-state characteristics in rough journal bearings, and the dynamic characteristics of hydrodynamic gas-lubricated microbearings were seldom reported in the research literature. Moreover, the statistical parameters (such as root mean square of asperity heights, surface slope, curvature, skewness, and kurtosis), which are conventionally applied to characterize surface roughness, vary with the sampling length and resolution of measuring equipment. A scale-invariant surface characterization should be considered. Hence, the analytical studies of surface roughness effect on dynamic characteristics of gas slider bearings with rarefaction coefficients in microfluidic engineering devices is motivated.

In this paper, the Weierstrass-Mandelbrot (W-M) fractal function is used to characterize the homogeneous surface roughness, and the Boltzmann slip correction model is applied to represent the rheological behavior of compressible rarefied gas film. The generalized Reynolds-type equation considering gas rarefaction, as well as roughness effect, is mathematically derived and solved by the partial derivative method and relaxation iteration algorithm. Bearing performances (including the load-carrying capacity, friction coefficient and corresponding attitude angle, dynamic stiffness, and damping coefficients) are presented and discussed in comparison with smooth surface bearings. The work is expected to elucidate the performance characteristics of gas microbearings with Poiseuille flow and random asperities, which is conducive to understand the fluid mechanisms of very low clearance gas films for microfluidics devices.

## 2. Characterization of Fractal Rough Surface

The distribution of asperity heights on the bearing surface consisting of long narrow ridges and valleys in engineering practice. A rough surface is a random system and the fractal geometry is introduced to characterize the random and multiscale topographies. Mandelbrot [29] initially developed the fractal theory by researching the coastal geomorphology in 1967. He found that the most machined surfaces can be constructed using Weierstrass-Mandelbrot (W-M) function with randomness, multiscale nature, self-similarity, and self-affine property. Unlike the traditional characterization parameters of surface roughness, fractal roughness parameters are independent of scan lengths and provide all the surface topography information of rough profiles. The W-M function that explicitly expressed the homogeneous rough surface in self-acting gas-lubricated journal bearings is given by

(1) $$\begin{array}{l} h_{r}(x,y)=L{\left(\frac{G}{L}\right)}^{D_{f}-2}\cdot \sqrt{\frac{\mathrm{In}\gamma }{M}}\cdot \sum_{m=1}^{M}\sum_{n=0}^{n_{\max}}\gamma^{(D_{f}-3)n}\times \\ \left(\cos\phi_{m,n}-\cos\left\{\frac{2\pi \gamma^{n}\sqrt{x^{2}+y^{2}}}{L}\cdot \cos\left[\tan^{-1}\left(\frac{y}{x}\right)-\frac{\pi m}{M}\right]+\phi_{m,n}\right\}\right) \end{array}$$

where *h_r_*(*x*,*y*) is the height of rough surface, *x* and *y* are the measure distances in the vertical and horizontal position, respectively. *L* is the sampling length of the profile of surface. *D_f_* is the fractal dimension, varying from 2 to 3 in three-dimensional surface topography. *G* is the scaling constant that relates to the roughness profile. *γ* is the scaling parameter (*γ* > 1), which determines the spectral density, *γ* is equal to 1.5 for a Gaussian and isotropic surface. *M* is the number of overlapped ridges on the surface, *m* and *n* are the frequency index, *n*_max_ = int[log(*L*/*L_s_*)/log*γ*], *φ_m_*_,*n*_ is the random phase, *L_s_* is the cut-off length that depends on cut-off wavelength of resolution in measuring machines.

**Figure 1:**
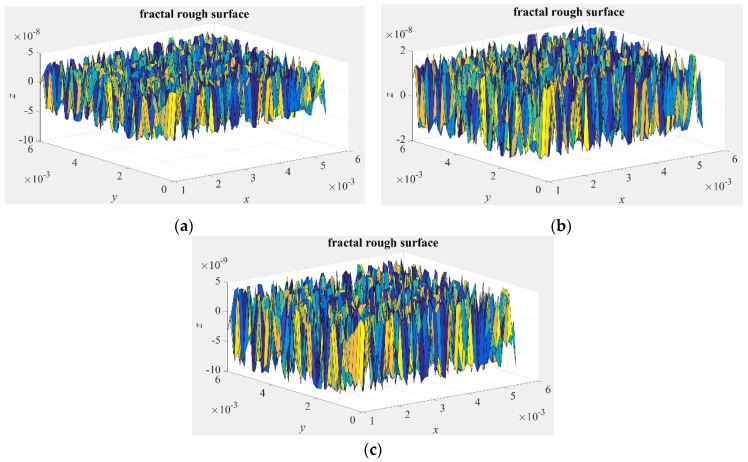
Simulation of a three-dimensional fractal surface topography for different fractal dimensions. (**a**) *D_f_* = 2.2, and *G* = 1 × 10^−10^ m; (**b**) *D_f_* = 2.3, and *G* = 1 × 10^−10^ m; (**c**) *D_f_* = 2.4, and *G* = 1 × 10^−10^ m.

The values of asperity heights can be changed by the fractal dimension *D_f_*, comparisons of the distributions of asperity heights for different *D_f_* are illustrated in Figure 1. It is seen that the heights of rough surfaces increase as the fractal dimension decreases.

**Figure 2:**
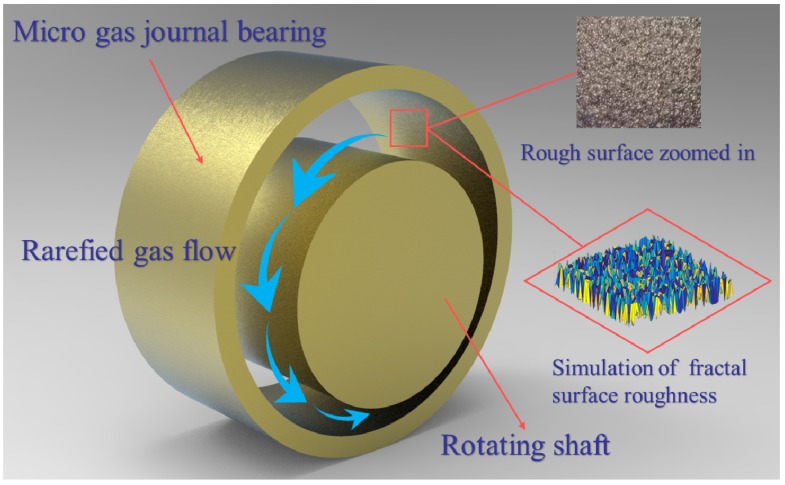
Schematic of a rough-surface gas-lubricated journal microbearings.

In the three-dimensional isotropic roughness type, the schematic presentation of rough gas microbearing is plotted in Figure 2.

## 3. Numerical Model and Solution Method

**Figure 3:**
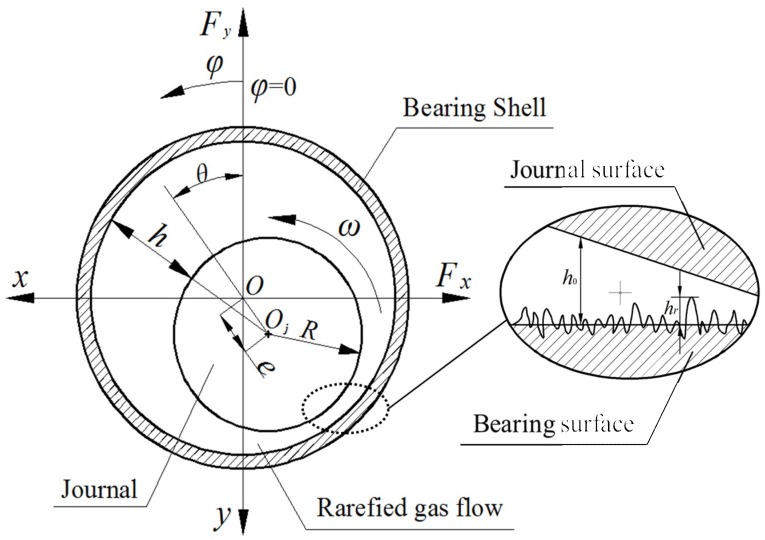
Geometrical configuration of the micro gas-lubricated journal bearing with rough surface.

In the current analysis, rarefied gas flow between the surfaces of bearing and journal is treated as isothermal, laminar flow with uniform viscosity. and elastic deformation of bearing surface is not considered. The Boltzmann slip correction is used to model the gas rarefaction effect at the gas-solid interface for arbitrary Knudsen numbers. The physical configuration of micro gas bearing with rough surface is shown in Figure 3. The modified Reynolds equation incorporating gas rarefaction and surface roughness effects in non-dimensional form appears as

(2) $$\frac{\partial }{\partial \varphi }\left(QPH^{3}\frac{\partial P}{\partial \varphi }\right)+\frac{\partial }{\partial \lambda }\left(QPH^{3}\frac{\partial P}{\partial \lambda }\right)=\Lambda \frac{\partial (PH)}{\partial \varphi }+2\Lambda \frac{\partial (PH)}{\partial T}$$

where *P* = *p*/*p_a_*, *H* = *h*/*c_b_*, *φ* = *x*/*R*, *λ* = *z*/*R* are the dimensionless gas film pressure, the dimensionless gas film thickness, and the coordinates in the circumferential and axial direction, *p_a_* is the ambient pressure, *c_b_* is the radius clearance, *R* is the radius of journal, *p* is the local gas pressure, *h* is the clearance spacing of ultra-thin gas film, *ε* is the eccentricity ratio and *ε* = *e*/*c_b_*, *e* is the eccentricity. *Λ* = 6*μωR*^2^/(*p_a_c_b_*^2^) is the gas bearing number, *μ* is the viscosity coefficient, *ω* is the rotating angular velocity of journal, *T* is the dimensionless time.

For the slip correction factors in rarefied gas flows under ultra-low spacing, the dimensionless Poiseuille flow rate between rotor and bearing surfaces with the gas thickness *h* is given as [30]

(3) $$Q_{P}=\frac{\frac{1}{2\mu }\frac{\partial p}{\partial x}h^{3}\left(b\cdot D^{c}+\frac{a\sqrt{\pi }}{2D}+\frac{1}{6}\right)}{\frac{-h^{3}}{2\mu D}\cdot \frac{\partial p}{\partial x}}=b\cdot D^{c+1}+\frac{a\sqrt{\pi }}{2}+\frac{D}{6}$$

The Poiseuille flow rate ratio *Q* appears as

(4) $$Q=\frac{Q_{P}}{Q_{continuum}}=\frac{b\cdot D^{c+1}+\frac{a\sqrt{\pi }}{2}+\frac{D}{6}}{\frac{D}{6}}=1+\frac{3a\sqrt{\pi }}{D}+6b\cdot D^{c}$$

where the inverse Knudsen number $D=\frac{\sqrt{\pi }}{2K_{n}}$, the three adjustable coefficients *a* = 0.01807, *b* = 1.35355 and *c* = −1.17468. The Boltzmann Poiseuille flow rate ratio [31] *Q* is expressed as

(5) $$Q=1+0.10842K_{n}+9.3593/K_{n}{}^{-1.17468}$$

Here the lubricant film thickness *h* is the sum of nominal smooth film thickness *h*_0_ and random roughness *h_r_* measured from the nominal smooth height.

(6) $$\begin{array}{l} h=h_{0}+h_{r}=c_{b}(1+\varepsilon \cos\varphi )+L{\left(\frac{G}{L}\right)}^{D_{f}-2}\cdot \sqrt{\frac{\mathrm{In}\gamma }{M}}\cdot \sum_{m=1}^{M}\sum_{n=0}^{n_{\max}}\gamma^{(D_{f}-3)n}\times \\ \left(\cos\phi_{m,n}-\cos\left\{\frac{2\pi \gamma^{n}\sqrt{x^{2}+y^{2}}}{L}\cdot \cos\left[\tan^{-1}\left(\frac{y}{x}\right)-\frac{\pi m}{M}\right]+\phi_{m,n}\right\}\right) \end{array}$$

As in the steady state, the transient term ∂(*PH*)/∂*T* of Equation (2) can be ignored, the static dimensionless modified Reynolds equation can be gained as

(7) $$\frac{\partial }{\partial \varphi }\left(QPH^{3}\frac{\partial P}{\partial \varphi }\right)+\frac{\partial }{\partial \lambda }\left(QPH^{3}\frac{\partial P}{\partial \lambda }\right)=\Lambda \frac{\partial (PH)}{\partial \varphi }$$

In order to obtain the aerodynamic performance of gas microbearings, the modified Reynolds equation for homogeneous surface roughness and gas rarefaction should be solved numerically. Equation (2), governing the gas pressure distribution of the bearing, is a nonlinear two-dimensional partial differential equation (PDE). It is difficult to get its analytical solution. So, the partial derivative method [32,33], with a relaxation iteration algorithm, is employed to ensure a reasonable and efficient solution. By introducing the mathematical transformation *PH* = *S*, (*PH*)^2^ = *S*^2^ = *Π*, Equation (2) can be converted to the ellipse-type partial differential equation −∇⋅(c∇u)+au=f in the following form:

(8) $$\begin{array}{l} -(\frac{\partial^{2}\Pi }{\partial \varphi^{2}}+\frac{\partial^{2}\Pi }{\partial \lambda^{2}})+\frac{2\Pi }{H}(\frac{\partial^{2}H}{\partial \varphi^{2}}+\frac{\partial^{2}H}{\partial \lambda^{2}})+\frac{2\Pi }{QH}(\frac{\partial Q}{\partial \varphi }\frac{\partial H}{\partial \varphi }+\frac{\partial Q}{\partial \lambda }\frac{\partial H}{\partial \lambda })=\\ -\frac{1}{H}(\frac{\partial H}{\partial \varphi }\frac{\partial \Pi }{\partial \varphi }+\frac{\partial H}{\partial \lambda }\frac{\partial \Pi }{\partial \lambda })+\frac{1}{Q}(\frac{\partial Q}{\partial \varphi }\frac{\partial \Pi }{\partial \varphi }+\frac{\partial Q}{\partial \lambda }\frac{\partial \Pi }{\partial \lambda })-\frac{2\Lambda }{QH}\frac{\partial S}{\partial \varphi }-\frac{4\Lambda }{QH}\frac{\partial S}{\partial T} \end{array}$$

The pressure boundary conditions for the Reynolds equation are:

(9) $$\left\{ \begin{array}{l} P|{}_{\varphi ,\lambda =\pm \frac{B}{2R}}=1,\\ P|{}_{\varphi =0,\lambda }=P|{}_{\varphi =2\pi ,\lambda },\\ \frac{\partial P}{\partial \varphi }|{}_{\varphi =0,\lambda }=\frac{\partial P}{\partial \varphi }|{}_{\varphi =2\pi ,\lambda } \end{array}\right.$$

where *B* is the bearing width.

The non-dimensional hydrodynamic gas film forces are obtained by integrating the film pressure acting on the microbearing along the both horizontal and vertical directions.

(10) $$\left\{ \begin{array}{l} {\bar{F}}_{x}=p_{a}R^{2}\int_{-\frac{B}{2R}}^{\frac{B}{2R}}\int_{0}^{2\pi }(P-1)\sin\varphi d\varphi d\lambda \\ {\bar{F}}_{y}=p_{a}R^{2}\int_{-\frac{B}{2R}}^{\frac{B}{2R}}\int_{0}^{2\pi }(P-1)\cos\varphi d\varphi d\lambda \end{array}\right.$$

The attitude angle *θ* of the gas-lubricated journal microbearing is calculated by

(11) $$\theta =\arctan\left(\frac{{\bar{F}}_{x}}{{\bar{F}}_{y}}\right)$$

The total non-dimensional load-carrying capacity is written as

(12) $$C_{L}=\frac{W}{p_{a}RB}=\frac{R}{B}\int_{-\frac{B}{2R}}^{\frac{B}{2R}}\int_{0}^{2\pi }(P-1)\cos\varphi d\varphi d\lambda$$

The non-dimensional skin friction coefficient on the journal surface can be computed by

(13) $$F_{b}=-\int_{-\frac{B}{2R}}^{\frac{B}{2R}}\int_{0}^{2\pi }\left(\frac{\Lambda }{6}\frac{1}{H}+\frac{H}{2}\frac{\partial P}{\partial \varphi }\right)d\varphi d\lambda$$

Suppose that the journal center whirls around its static equilibrium position with a small amplitude periodic motion under the perturbation frequency ratio *Ω*, the linear perturbation method is adopted for calculating the dynamic stiffness and damping coefficients. The steady-state position is indicated as (*ε*_0_,*θ*_0_), and its dynamic disturbance about (*ε*_0_,*θ*_0_) are denoted as *E* and *Θ*. The positions of eccentricity ratio *ε* and attitude angle *θ* of journal at a random position are represented by the static and dynamic components as follows:

(14) $$\left\{ \begin{array}{l} \varepsilon =\varepsilon_{0}+E=\varepsilon_{0}+E_{0}e^{i\Omega T}\\ \theta =\theta_{0}+\Theta =\theta_{0}+\Theta_{0}e^{i\Omega T} \end{array}\right.$$

where *E*_0_ and *Θ*_0_ are the small perturbation amplitude of journal eccentricity ratio and attitude angle in the complex field. The dimensionless perturbation frequency *Ω*, which is defined as the ratio of journal disturbance frequency *ν* to rotating angular velocity *ω* of journal, $i=\sqrt{-1}$.

Thus, the non-dimensional gas-film pressure and gas-film thickness of the gas microbearing can be expressed as

(15) $$\left\{ \begin{array}{l} P=P_{0}+Q_{gd}=P_{0}+{\tilde{P}}_{0}e^{i\Omega T}\\ H=H_{0}+H_{gd}=H_{0}+{\tilde{H}}_{0}e^{i\Omega T} \end{array}\right.$$

where ${\tilde{H}}_{0}=E_{0}\cos(\varphi -\theta_{0})+\varepsilon_{0}\Theta_{0}\sin(\varphi -\theta_{0})$, *P*_0_ is the static gas-film pressure and *H*_0_ is the static gas-film thickness. *Q_gd_*, *H_gd_* are the dynamic gas film pressure and gas film thickness, respectively. ${\tilde{P}}_{0}$ and ${\tilde{H}}_{0}$ are the perturbation magnitudes in terms of complex numbers for dynamic gas film pressure and gas film thickness.

Substituting Equation (15) into Equation (2), the generalized dynamic lubrication equation for molecular model and surface roughness can be derived as:

(16) $$\begin{array}{l} \frac{\partial }{\partial \varphi }(QP_{0}H_{0}{}^{3}\frac{\partial {\tilde{P}}_{0}}{\partial \varphi })+\frac{\partial }{\partial \lambda }(QP_{0}H_{0}{}^{3}\frac{\partial {\tilde{P}}_{0}}{\partial \lambda })+\frac{\partial }{\partial \varphi }(Q{\tilde{P}}_{0}H_{0}{}^{3}\frac{\partial P_{0}}{\partial \varphi })+\frac{\partial }{\partial \lambda }(Q{\tilde{P}}_{0}H_{0}{}^{3}\frac{\partial P_{0}}{\partial \lambda })+\\ \frac{\partial }{\partial \varphi }(3QP_{0}H_{0}{}^{2}{\tilde{H}}_{0}\frac{\partial P_{0}}{\partial \varphi })+\frac{\partial }{\partial \lambda }(3QP_{0}H_{0}{}^{2}{\tilde{H}}_{0}\frac{\partial P_{0}}{\partial \lambda })=\Lambda \frac{\partial }{\partial \varphi }(P_{0}{\tilde{H}}_{0}+{\tilde{P}}_{0}H_{0})+i2\Lambda \Omega (P_{0}{\tilde{H}}_{0}+{\tilde{P}}_{0}H_{0}) \end{array}$$

As mentioned in Reference [34], some variables are defined by

(17) $$\left\{ \begin{array}{l} P_{E}=\frac{\partial {\tilde{P}}_{0}}{\partial E_{0}},\\ P_{\theta }=\frac{1}{\varepsilon_{0}}\frac{\partial {\tilde{P}}_{0}}{\partial \Theta_{0}},\\ H_{E}=\frac{\partial {\tilde{H}}_{0}}{\partial E_{0}},\\ H_{\theta }=\frac{1}{\varepsilon_{0}}\frac{\partial {\tilde{H}}_{0}}{\partial \Theta_{0}} \end{array}\right.$$

After differentiating ${\tilde{P}}_{0}$ and ${\tilde{H}}_{0}$ in Equation (16) with respect to *E*_0_ and *Θ*_0_, and combining with some mathematical transformation, the resulting dynamic PDE equations are obtained for rough surface microbearings concerning the variables *P_E_*, *P_θ_*, *H_E_*, and *H_θ_*.

(18) $$\begin{array}{l} \frac{\partial }{\partial \varphi }(QP_{0}H_{0}{}^{3}\frac{\partial P_{E}}{\partial \varphi })+\frac{\partial }{\partial \lambda }(QP_{0}H_{0}{}^{3}\frac{\partial P_{E}}{\partial \lambda })+\frac{\partial }{\partial \varphi }(QP_{E}H_{0}{}^{3}\frac{\partial P_{0}}{\partial \varphi })+\frac{\partial }{\partial \lambda }(QP_{E}H_{0}{}^{3}\frac{\partial P_{0}}{\partial \lambda })+\\ \frac{\partial }{\partial \varphi }(\frac{\partial Q}{\partial E_{0}}P_{0}H_{0}{}^{3}\frac{\partial {\tilde{P}}_{0}}{\partial \varphi })+\frac{\partial }{\partial \lambda }(\frac{\partial Q}{\partial E_{0}}P_{0}H_{0}{}^{3}\frac{\partial {\tilde{P}}_{0}}{\partial \lambda })+\frac{\partial }{\partial \varphi }(\frac{\partial Q}{\partial E_{0}}{\tilde{P}}_{0}H_{0}{}^{3}\frac{\partial P_{0}}{\partial \varphi })+\\ \frac{\partial }{\partial \lambda }(\frac{\partial Q}{\partial E_{0}}{\tilde{P}}_{0}H_{0}{}^{3}\frac{\partial P_{0}}{\partial \lambda })+3QP_{0}H_{0}{}^{3}\frac{\partial P_{0}}{\partial \varphi }\frac{\partial }{\partial \varphi }(\frac{H_{E}}{H_{0}})+3QP_{0}H_{0}{}^{3}\frac{\partial P_{0}}{\partial \lambda }\frac{\partial }{\partial \lambda }(\frac{H_{E}}{H_{0}})+\\ 3\frac{\partial }{\partial \varphi }(\frac{\partial Q}{\partial E_{0}}P_{0}H_{0}{}^{2}{\tilde{H}}_{0}\frac{\partial P_{0}}{\partial \varphi })+3\frac{\partial }{\partial \lambda }(\frac{\partial Q}{\partial E_{0}}P_{0}H_{0}{}^{2}{\tilde{H}}_{0}\frac{\partial P_{0}}{\partial \lambda })+3\Lambda \frac{H_{E}}{H_{0}}\frac{\partial (P_{0}H_{0})}{\partial \varphi }=\\ \Lambda \frac{\partial }{\partial \varphi }(P_{0}H_{E}+P_{E}H_{0})+i2\Lambda \Omega (P_{0}H_{E}+P_{E}H_{0}) \end{array}$$

(19) $$H_{E}=\cos(\varphi -\theta_{0})$$

(20) $$\begin{array}{l} \frac{\partial }{\partial \varphi }(QP_{0}H_{0}{}^{3}\frac{\partial P_{\theta }}{\partial \varphi })+\frac{\partial }{\partial \lambda }(QP_{0}H_{0}{}^{3}\frac{\partial P_{\theta }}{\partial \lambda })+\frac{\partial }{\partial \varphi }(QP_{\theta }H_{0}{}^{3}\frac{\partial P_{0}}{\partial \varphi })+\frac{\partial }{\partial \lambda }(QP_{\theta }H_{0}{}^{3}\frac{\partial P_{0}}{\partial \lambda })+\\ \frac{\partial }{\partial \varphi }(\frac{\partial Q}{\partial \Theta_{0}}P_{0}H_{0}{}^{3}\frac{\partial {\tilde{P}}_{0}}{\partial \varphi })+\frac{\partial }{\partial \lambda }(\frac{\partial Q}{\partial \Theta_{0}}P_{0}H_{0}{}^{3}\frac{\partial {\tilde{P}}_{0}}{\partial \lambda })+\frac{\partial }{\partial \varphi }(\frac{\partial Q}{\partial \Theta_{0}}{\tilde{P}}_{0}H_{0}{}^{3}\frac{\partial P_{0}}{\partial \varphi })+\\ \frac{\partial }{\partial \lambda }(\frac{\partial Q}{\partial \Theta_{0}}{\tilde{P}}_{0}H_{0}{}^{3}\frac{\partial P_{0}}{\partial \lambda })+3QP_{0}H_{0}{}^{3}\frac{\partial P_{0}}{\partial \varphi }\frac{\partial }{\partial \varphi }(\frac{H_{\theta }}{H_{0}})+3QP_{0}H_{0}{}^{3}\frac{\partial P_{0}}{\partial \lambda }\frac{\partial }{\partial \lambda }(\frac{H_{\theta }}{H_{0}})+\\ 3\frac{\partial }{\partial \varphi }(\frac{\partial Q}{\partial \Theta_{0}}P_{0}H_{0}{}^{2}{\tilde{H}}_{0}\frac{\partial P_{0}}{\partial \varphi })+3\frac{\partial }{\partial \lambda }(\frac{\partial Q}{\partial \Theta_{0}}P_{0}H_{0}{}^{2}{\tilde{H}}_{0}\frac{\partial P_{0}}{\partial \lambda })+3\Lambda \frac{H_{\theta }}{H_{0}}\frac{\partial (P_{0}H_{0})}{\partial \varphi }=\\ \Lambda \frac{\partial }{\partial \varphi }(P_{0}H_{\theta }+P_{\theta }H_{0})+i2\Lambda \Omega (P_{0}H_{\theta }+P_{\theta }H_{0}) \end{array}$$

(21) $$H_{\theta }=\sin(\varphi -\theta_{0})$$

According to the coordinate system at the bearing midplane as illustrated in Figure 3, by simultaneously solving the nonlinear Equations (18)–(21) using the partial derivative method with iteration procedure, the dynamic stiffness coefficients *K_ij_* and dynamic damping coefficients *D_ij_* of gas journal microbearing for fractal rough surface can be calculated by the following formula:

(22) $$\left\{ \begin{array}{l} -\frac{R}{B}\iint_{A}P_{E}\cos\varphi d\varphi d\lambda =K_{y\varepsilon }+i\Omega D_{y\varepsilon }\\ \frac{R}{B}\iint_{A}P_{E}\sin\varphi d\varphi d\lambda =K_{x\varepsilon }+i\Omega D_{x\varepsilon }\\ -\frac{R}{B}\iint_{A}P_{\theta }\cos\varphi d\varphi d\lambda =K_{y\theta }+i\Omega D_{y\theta }\\ \frac{R}{B}\iint_{A}P_{\theta }\sin\varphi d\varphi d\lambda =K_{x\theta }+i\Omega D_{x\theta } \end{array}\right.$$

The dynamic coefficients in the Cartesian coordinate system are given by the transformation matrix *A*.

(23) $$\left\{ \begin{array}{l} K_{ij}=\left(\begin{array}{cc} K_{xx} & K_{xy}\\ K_{yx} & K_{yy} \end{array}\right)=A\left(\begin{array}{cc} K_{x\varepsilon } & K_{x\theta }\\ K_{y\varepsilon } & K_{y\theta } \end{array}\right)\\ D_{ij}=\left(\begin{array}{cc} D_{xx} & D_{xy}\\ D_{yx} & D_{yy} \end{array}\right)=A\left(\begin{array}{cc} D_{x\varepsilon } & D_{x\theta }\\ D_{y\varepsilon } & D_{y\theta } \end{array}\right) \end{array}\right.,A=\left(\begin{array}{cc} -\sin\theta_{0} & -\cos\theta_{0}\\ \cos\theta_{0} & -\sin\theta_{0} \end{array}\right)$$

It was confirmed that the dynamic characteristics of the gas journal bearing can greatly affect the critical speed, unbalance response, and instability threshold of a hydrodynamic gas-lubricated bearing-rotor system after long-term research and practice. The comprehensive analysis of dynamic coefficients is important in the design of gas microbearings in MEMS applications, the key findings presented in the next section will reveal some insights into the ultra-thin gas film lubrication problems.

## 4. Results and Discussion

On the basis of fractal geometry theory and the Boltzmann slip correction factor, the combined effects of gas rarefaction and surface roughness on the static and dynamic characteristics of ultra-thin film gas lubrication in journal microbearings are investigated in detail. The pressure distribution, load carrying capacity, friction coefficient and attitude angles of bearing, dynamic stiffness and damping properties are analyzed concerning the fractal dimension and bearing geometric parameters in this section, the primary design parameters shown in Figure 3 are *R* = 1mm, *B* = 200 µm, *c* = 1 µm, *p_a_* = 1.033 × 10^5^ N/m^2^, and the aspect ratio is *B*/*D* = 0.1.

### 4.1. Steady-State Film Pressure

**Figure 4:**
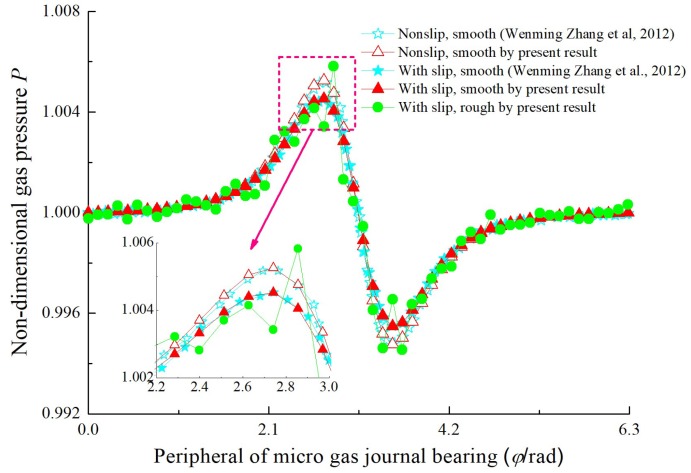
Comparison of dimensionless gas film pressure with Zhang et al. [20].

To verify the correctness of the developed theoretical model and program code employed in this paper, the non-dimensional pressure profile for the middle cross-section along the sliding direction obtained by the current solution is compared with Zhang et al. in Reference [22]. As indicated in Figure 4, the simulation results are in close agreement with the numerical predictions reported by Zhang et al. for *ε* = 0.7, *B*/*D* = 0.075, *c* = 12 μm, *ω* = 5 × 10^5^ rpm. It can also be seen that the pressure randomly fluctuates when the effect of surface roughness is considered.

**Figure 5:**
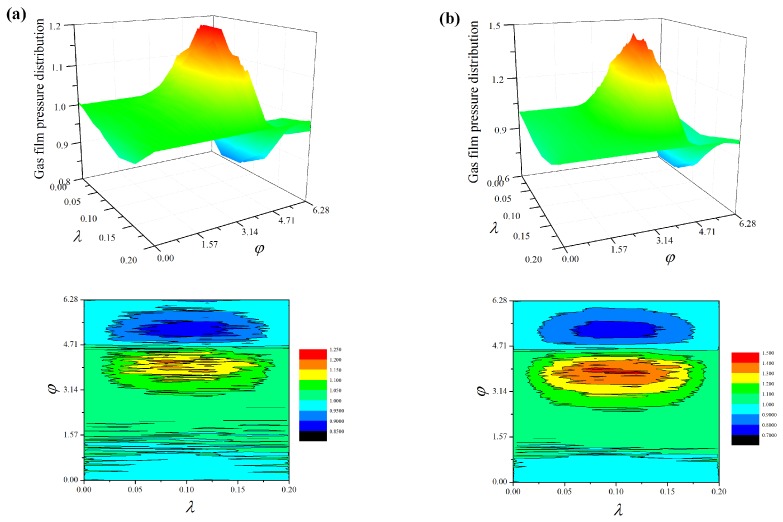

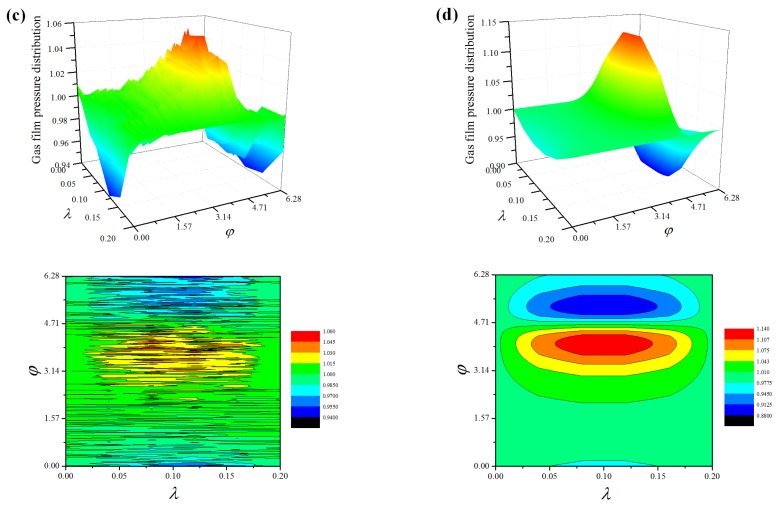
Pressure distributions and contour plots of the gas-lubricated microbearing for different bearing numbers and eccentricity ratios: (**a**) *ε* = 0.6, *Λ* = 20, *D_f_* = 2.3, and *G* = 1 × 10^−10^ m, (**b**) *ε* = 0.6, *Λ* = 60, *D_f_* = 2.3, and *G* = 1 × 10^−10^ m, (**c**) *ε* = 0.3, *Λ* = 20, *D_f_* = 2.3, and *G* = 1 × 10^−10^ m, and (**d**) *ε* = 0.6, *Λ* = 20, smooth.

The influence of bearing number *Λ*, eccentricity ratio *ε*, and surface roughness on pressure distributions *P* is shown in Figure 5. An important observation exhibited by Figure 4a,b is that the maximum gas film pressure becomes larger as *Λ* increases for fixed fractal parameters *G* and *D*. This phenomenon can be explained by the enhanced aerodynamic effect, which indicates that higher angular velocity of the journal dilutes the pressure diffusion. In Figure 4a,c, increasing the eccentricity ratio *ε* leads to the higher pressure distribution and magnitude of pressure fluctuations decrease. In comparison with the smooth surface case, the roughness effect increases the pressure profile for the rough surface at the same bearing number and eccentricity ratio, and the random surface roughness makes the pressure distribution across the entire lubricating film unpredictable. The contour plots show more clearly the detail and variation of pressure over the rough bearing surfaces. At greater bearing number *Λ* the influence of surface roughness decreases and the contour lines approach that of the smooth case, the fluctuations of the pressure contour plots are significant with the increase of fractal roughness in gas bearing.

### 4.2. Load-Carry Capacity and Friction Coefficient

**Figure 6:**
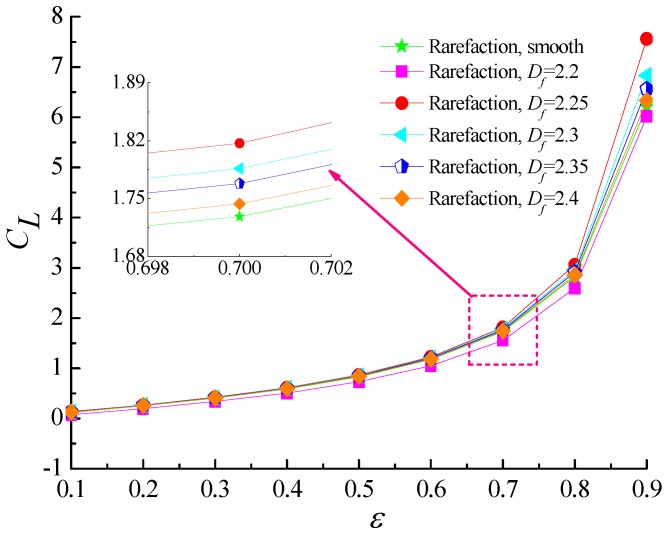
Non-dimensional load capacity versus eccentricity ratio for different fractal dimensions at *Λ* = 20, *G* = 1 × 10^−11^.

**Figure 7:**
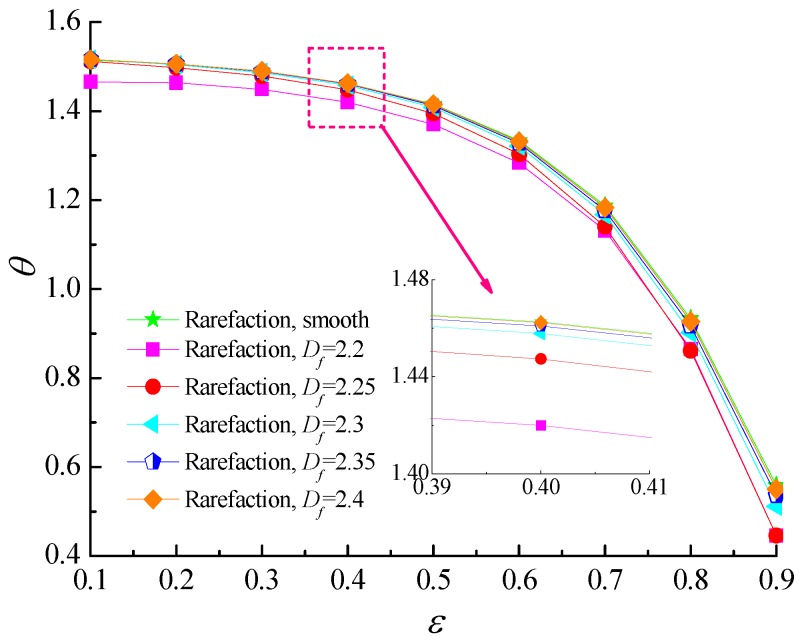
Attitude angle versus eccentricity ratio for different fractal dimensions at *Λ* = 20, *G* = 1 × 10^−11^.

Figure 6 and Figure 7 predict the variation of non-dimensional load carrying capacity and attitude angle as a function of eccentricity ratio for five different values of fractal dimension (*D_f_* = 2.2, 2.25, 2.3, 2.35, 2.4). As the eccentricity ratio increases, which denotes the film thickness is thinner, the load capacity increases monotonically. The higher self-affine fractal dimensions yield the smaller roughness heights distribution on rough surfaces, and it is noted that the load carrying capacity is increased gradually when compared with the smooth bearing. The reason is that the increasing values of roughness heights reduces the sidewise leakage of airflow and the flow is restricted by the surface asperities. However, as the fractal dimension decreases further for *D_f_* = 2.2, the load carrying capacity tends to decline under this condition. This is because the minimum air film clearance between rotor and bearing may become too small so that the surface roughness effect which increases the load-carrying capacity is weaker than the gaseous rarefaction effects which reduces the dimensionless load capacity, thus causing a decrease in the bearing load capacity. The attitude angle *θ* is found to decrease with the growing eccentricity ratio. The decrease in attitude angle is more accentuated for a rougher surface as compared to a nominally smooth surface.

**Figure 8:**
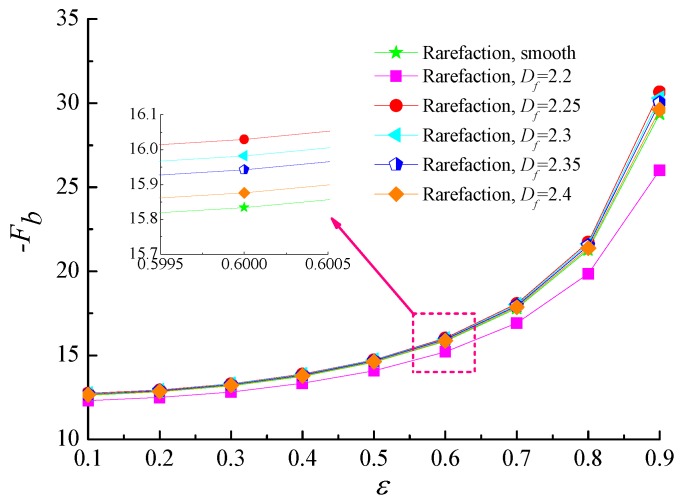
Static friction coefficient versus eccentricity ratio for different fractal dimensions at *Λ* = 20, *G* = 1 × 10^−11^.

The variation of friction coefficient with the eccentricity ratio for different values of fractal dimension with fixed values of *Λ* = 20 and *G* = 1 × 10^−11^ is depicted in Figure 8. It can be seen that friction coefficients monotonically increase as ε increases. Although the contact area between the rarefied gas flow and the surface asperities is larger when the homogeneous surface roughness becomes more and more obvious, the static friction coefficients show a slightly more gradual increase with fractal dimension. As the fractal dimension *D_f_* is equal to 2.2, the friction coefficient is lower than that of smooth surface for the same reason that the gaseous rarefaction effect is more pronounced and thus friction coefficient drops.

**Figure 9:**
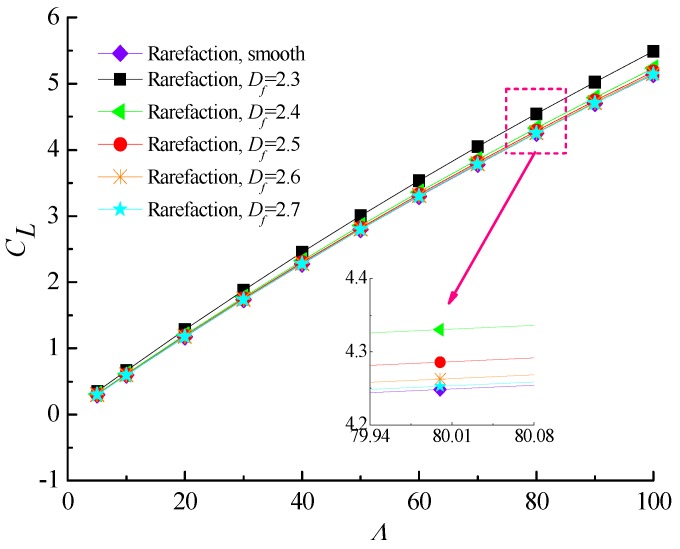
Non-dimensional load capacity versus bearing number for different fractal dimensions at *ε* = 0.6, *G* = 1 × 10^−10^.

**Figure 10:**
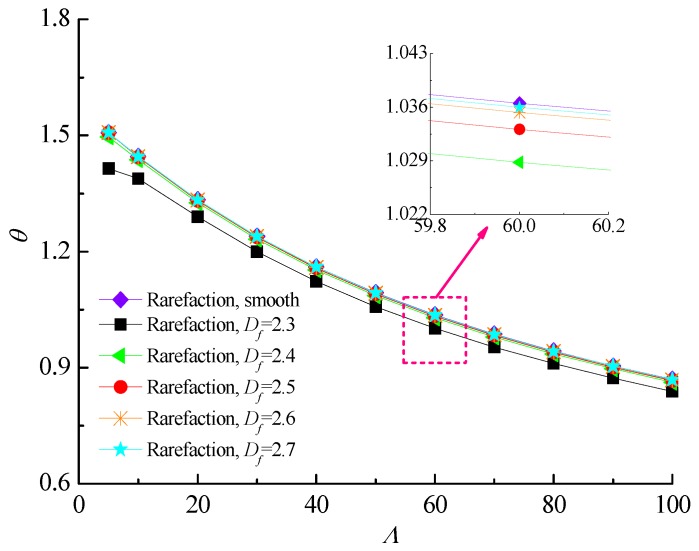
Attitude angles versus bearing number for different fractal dimensions at *ε* = 0.6, *G* = 1 × 10^−10^.

**Figure 11:**
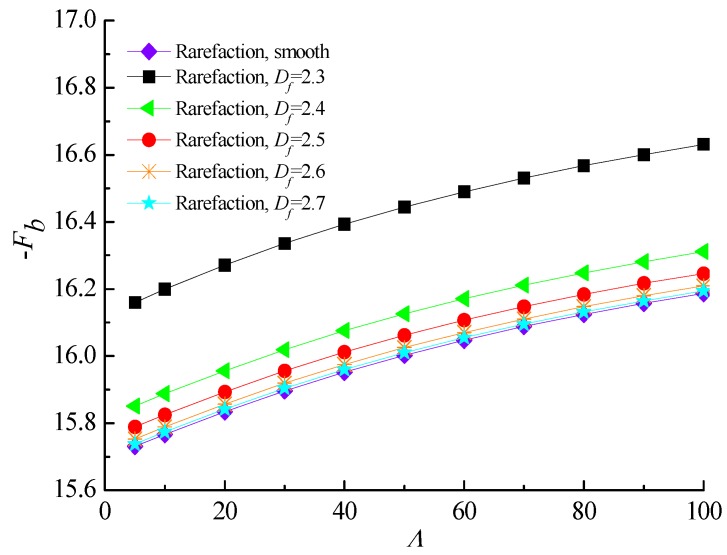
Static friction coefficient versus bearing number for different fractal dimensions at *ε* = 0.6, *G* = 1 × 10^−10^.

Figure 9 and Figure 10 describe the effect of increasing bearing number on the load carrying capacity and attitude angle with various fractal dimensions for *ε* = 0.6 and *G* = 1 × 10^−10^. It is found that increasing the values of the bearing number from *Λ* = 3 up to *Λ* = 100 increases the carrying capacity and reduces the corresponding attitude angle of gas journal microbearing. The surface roughness effect in aerodynamic lubrication enhances the load capacity as compared with the smooth-bearing case, especially for the bearing operating at high bearing number. Figure 11 shows the comparison of the static friction coefficients with bearing number for different fractal dimensions. The friction coefficients exhibit a near-linear increasing trend with increasing *Λ*, while the increase extent in static friction coefficient is even higher at smaller *D_f_* values. Consequently, the strengthened gas-lubricated hydrodynamic effect and the bearing surface with roughness undulations have a significant influence on the skin friction at the interface.

### 4.3. Dynamic Stiffness and Damping Coefficients

**Figure 12:**
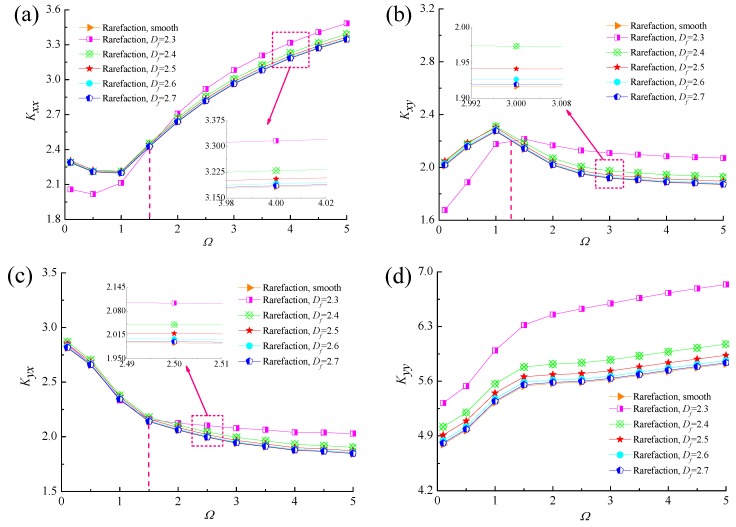
Effect of perturbation frequency on dynamic stiffness coefficients with different fractal dimensions. (**a**) *K_xx_* vs. *Ω*; (**b**) *K_xy_* vs. *Ω*; (**c**) *K_yx_* vs. *Ω*; (**d**) *K_yy_* vs. *Ω*.

**Figure 13:**
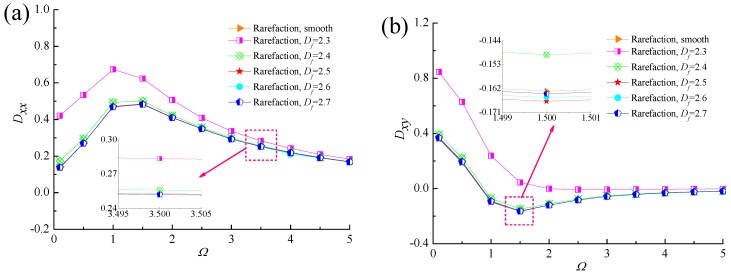

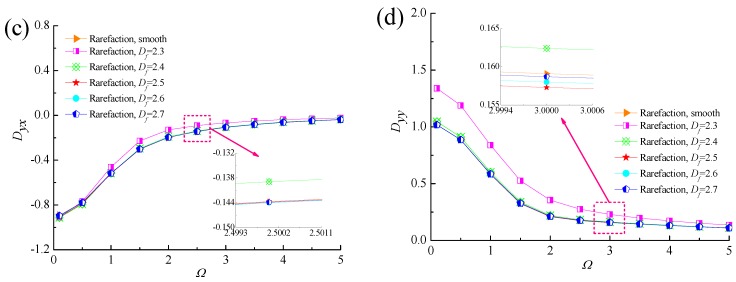
Effect of perturbation frequency on dynamic damping coefficients with different fractal dimensions. (**a**) *D_xx_* vs. *Ω*; (**b**) *D_xy_* vs. *Ω*; (**c**) *D_yx_* vs. *Ω*; (**d**) *D_yy_* vs. *Ω*.

The variation of dynamic stiffness and damping coefficients with dimensionless perturbation frequency *Ω* for different values of fractal dimensions is plotted in Figure 12 and Figure 13. The principal stiffness coefficients *K_xx_* and *K_yy_* increases as the perturbation frequency increases and the *K_yy_* is much greater than *K_xx_* because of the rarefied gas lubricating film supports the weight of journal in the vertical direction. The cross-couple stiffness *K_xy_* increases at first, then decreases slightly with the growth of *Ω*, while *K_yx_* decreases quickly at lower perturbation frequencies. Furthermore, the enhanced dynamic stiffness coefficients are seen for the rough bearing surface as compared to that of smooth bearing case. When *Ω* > 2, all the dynamic stiffness coefficients of micro gas-lubricated journal bearing at the fractal dimension *D_f_* = 2.3 are obviously greater than other rough surfaces. This is mainly due to the fact that the larger asperity heights lead to an increased Poiseuille flow component along the sliding direction and the side flow suffers the constriction resistance caused by homogeneous surface roughness. The influence of perturbation frequency on dynamic damping coefficients for different fractal roughness parameters can be observed from Figure 13. The principal damping coefficient *D_xx_* first increases with increasing dimensionless perturbation frequency, reaches a maximum, then starts to decline slowly. The absolute values of the cross-coupling terms of damping coefficients *D_xy_* and *D_yx_* decrease quickly at low *Ω*, then approaches to zero. The result show that the damping coefficients *D_xx_*, *D_yx_* increase with the increase in the isotropic and homogeneous roughness heights of gas slider bearing surface, and the *D_xy_* and *D_yy_* are first decreases and then dramatically increases as the fractal dimension *D_f_* decreases, whereas the difference in damping coefficients of rough and smooth cases appear to converge at higher values of *Ω*.

**Figure 14:**
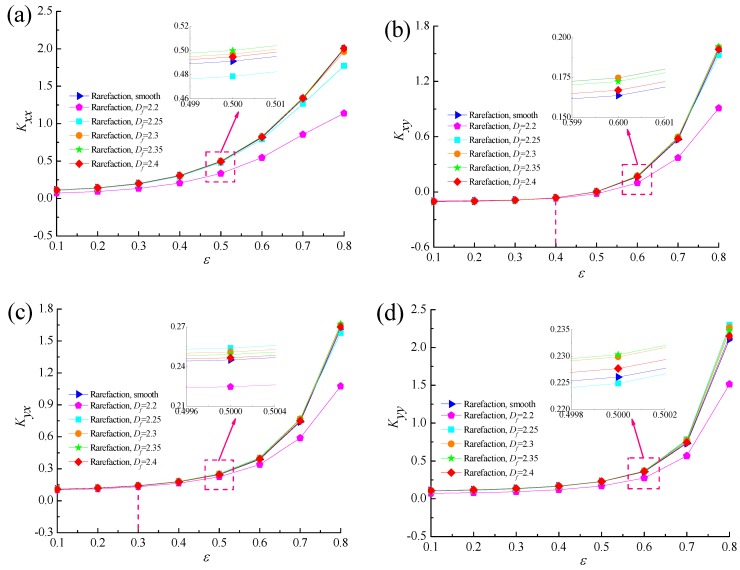
Effect of eccentricity ratio on dynamic stiffness coefficients with different fractal dimensions. (**a**) *K_xx_* vs. *ε*; (**b**) *K_xy_* vs. *ε*; (**c**) *K_yx_* vs. *ε*; (**d**) *K_yy_* vs. *ε*.

**Figure 15:**
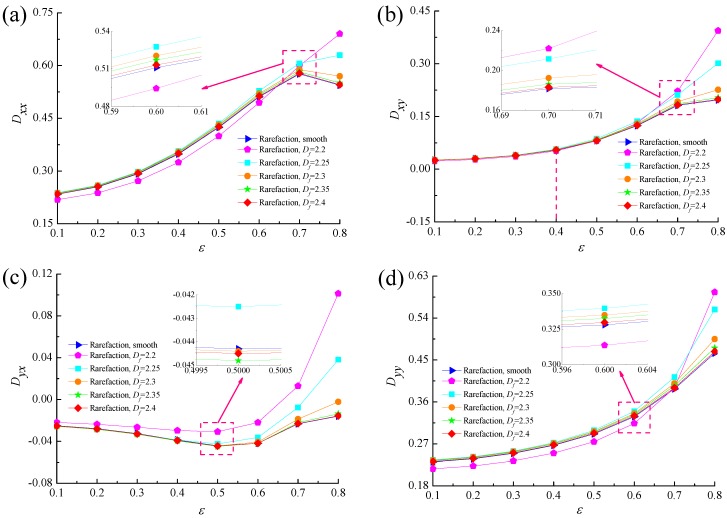
Effect of eccentricity ratio on dynamic damping coefficients with different fractal dimensions. (**a**) *D_xx_* vs. *ε*; (**b**) *D_xy_* vs. *ε*; (**c**) *D_yx_* vs. *ε*; (**d**) *D_yy_* vs. *ε*.

Figure 14 and Figure 15 display the relationship between the dynamic coefficients of gas microbearing and eccentricity ratio with various fractal dimensions. It is found that the dynamic stiffness coefficients increase as the eccentricity ratio increases for fixed values of *Λ* = 20 and *G* = 1 × 10^−11^. The higher *ε* corresponds to the thinner gas film thickness, which results in the increase of Knudsen number *K_n_*. With the growth of the fractal surface roughness, the stiffness coefficients first increase gradually, then decrease significantly at the same *ε*. As illustrated in Figure 15, the damping coefficients increase marginally as the eccentricity ratio increases and the effect of fractal dimension on damping coefficient *D_xy_* is negligible at lower eccentricity ratios. The damping coefficients become more sensitive to surface roughness at higher eccentricity ratios about *ε* > 0.7. It can be seen that increasing the random roughness heights increase the effect of gas rarefaction in small spacing.

**Figure 16:**
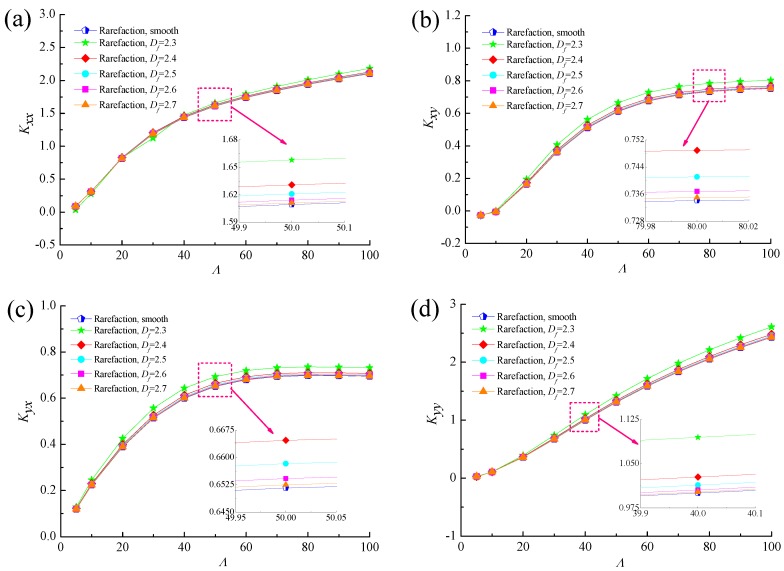
Effect of bearing number on dynamic stiffness coefficients with different fractal dimensions. (**a**) *K_xx_* vs. *Λ*; (**b**) *K_xy_* vs. *Λ*; (**c**) *K_yx_* vs. *Λ*; (**d**) *K_yy_* vs. *Λ*.

**Figure 17:**
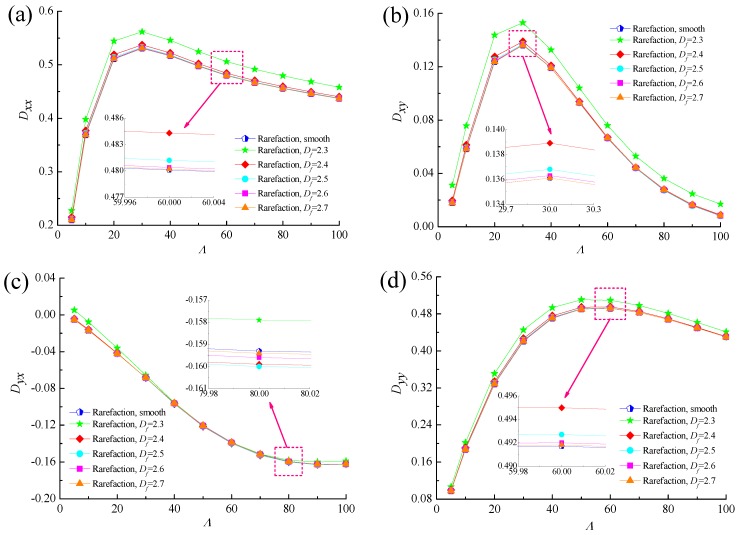
Effect of bearing number on dynamic damping coefficients with different fractal dimensions. (**a**) *D_xx_* vs. *Λ*; (**b**) *D_xy_* vs. *Λ*; (**c**) *D_yx_* vs. *Λ*; (**d**) *D_yy_* vs. *Λ*.

Figure 16 and Figure 17 show the comparisons of the dynamic characteristics between rough and smooth bearing surfaces with different bearing numbers for the fixed values of *ε* = 0.6 and *G* = 1 × 10^−10^. Increment of the bearing number means the larger operating conditions. The principal stiffness *K_yy_* is near proportionally dependent on *Λ* and dynamic stiffness coefficients *K_xx_*, *K_xy_* and *K_yx_* increase gradually with increasing bearing number. It is also observed that the increase in the dynamic stiffness coefficients is more accentuated for the fractal roughness surface as compared to the smooth surface bearing with the enhanced aerodynamic effect in gas journal bearings. The damping coefficients exhibit similar trends to the fractal dimension, namely the principal damping *D_xx_*, *D_yy_* and cross-couple damping *D_xy_* for rough bearing surface become larger than the ones in the smooth bearing, whereas the roughness effect is rather marginal in the case of the cross-couple damping *D_yx_* in Figure 17c. The damping coefficients *D_xx_*, *D_yy_* and *D_xy_* increase quickly at first and then decreased, while the damping coefficient *D_yx_* decreases with increasing bearing number. Therefore, the dynamic stiffness and damping characteristics of gas microbearings with isotropic and homogeneous roughness show relatively high values since the aerodynamic effect of the bearing is enhanced.

## 5. Conclusions

The coupled effects of gas rarefaction and surface roughness on the static and dynamic characteristics of gas microbearings are studied using fractal geometry theory and Boltzmann model for Poiseuille flow. Based on the results mentioned in previous section, the following conclusions have been drawn:

1. Surface roughness and gaseous rarefaction effects are of great importance to the lubrication performance of gas journal microbearings. At small asperity height distributions, the gas film pressure distributions increase for higher bearing numbers and eccentricity ratios, consequently leads to an increment of load-carrying capacity.

2. The attitude angle increases with the larger fractal dimensions compared with smooth surface, whereas the skin friction coefficient yields a reversed trend because of the long narrow ridges and furrows impose a series of constrictions on gas lubricant in the sliding direction.

3. The principal stiffness coefficients increase with the increase in dimensionless perturbation frequency and all the stiffness coefficients become larger and larger at higher eccentricity ratios and bearing numbers. The damping coefficients increase as the eccentricity ratio increases and the principal damping can be first magnified and then diminished with increasing bearing number.

4. The stiffness coefficients increase as the fractal dimension decreases at the same perturbation frequency and bearing number, while the stiffness coefficients first increase and then decrease for increasing eccentricity ratio. The damping coefficients increase with decreasing fractal dimension except for the *D_yx_* at large bearing number values.

5. It seems that the fractal rough surface does not merely increases the steady-state and dynamic performance of gas microbearings. When the degree of rarefaction effect is more pronounced, the static load capacity and dynamic coefficients decrease quickly for larger values of distribution of asperity heights.
